# Surgery as first-line treatment for prolactinoma? Discussion of the literature and results of a consecutive series of surgically treated patients

**DOI:** 10.1007/s10143-023-02033-0

**Published:** 2023-05-30

**Authors:** Malte Ottenhausen, Jens Conrad, Lea-Marie Wolters, Florian Ringel

**Affiliations:** Department of Neurosurgery, University Medical Center Mainz, Langenbeckstr. 1, 55131 Mainz, Germany

**Keywords:** Pituitary adenoma, Prolactinoma, Transsphenoidal, Endoscopic

## Abstract

Initial treatment for prolactinoma is usually conservative with dopamine agonists. However, the duration of treatment is often lifelong and can be associated with significant side effects. Surgical outcomes are usually favorable and treatment complications low, raising the question whether surgical therapy should be included earlier in the treatment of prolactinoma. The aim of this study was to analyze the outcome of surgical resection of prolactinomas at our institution, to compare it with other published surgical and conservative series and to discuss the role of surgery in modern prolactinoma therapy. The authors reviewed a database of single-center consecutively operated prolactinoma cases and analyzed the extent of resection (EOR), endocrinological and neurological outcomes, and complications. Thirty patients were analyzed. Mean patient age was 37.2 ± 15.5 years (range 16–76) and consisted of 17 (56.7%) females and 13 (43.3%) males. Twenty-one patients (70%) failed medical therapy, the main reasons being intolerable side effects in 11 cases (52.4%) and insufficient response in 10 cases (47.6%). Nine patients (30%) received no medical treatment prior to surgery, of which five (55.6%) were operated because of pituitary apoplexy, two (22.2%) because of acute visual deterioration and two (22.2%) refused medical treatment and opted for surgery as first-line treatment. Of the 30 operated tumors, 56.7% (*n* = 17) were microadenomas, 30% (*n* = 9) were macroadenomas (≥ 10 mm), and 13.3% (*n* = 4) were giant adenomas (≥ 40 mm). GTR was achieved in 75% (*n* = 21) of cases. The overall remission rate was 63.3%. MRI showed a residual tumor in seven patients (25%), typically with invasive growth. Postoperative CSF leaks did not occur. Mean follow-up was 34.9 ± 60.3 months (range 0–246 months). Endocrine remission was defined as a morning fasting basal PRL level of 22.3 < ng/mL and measured at the last available follow-up. Postoperative Prolactine levels were missing in three patients. Our analysis describes a highly selected sample with a disproportionate number of larger, invasive tumors and emergency cases. Nevertheless, the results are satisfactory and comparable with other published series. The consistently good results of transphenoidal surgery, especially for microprolactinomas, have led to a greater acceptance of surgery in the treatment of prolactinomas in recent years. The timing of surgery in each individual case must be determined by a multidisciplinary team to ensure the best possible outcome.

## Introduction

Prolactinomas or lactotrophic adenomas are the most common pituitary adenomas, accounting for approximately 50% of all pituitary adenomas and are characterized by excessive prolactin secretion (levels > 150–200 ng/mL). These high levels of prolactin can cause decreased fertility, decreased energy and libido, and galactorrhea in men and premenopausal women. Headache is a common symptom in men, pre- and postmenopausal women while hypogonadism is seen exclusively in men, and oligo- or amenorrhea is seen exclusively in premenopausal women [1]. Visual field deficits and visual acuity deficits can result from compression of the optic nerves and chiasm.

While elevated prolactin levels (hyperprolactinemia) can also be caused by other pituitary adenomas through increased intrasellar pressure, known as the stalk effect, or by hypothalamic dysfunction, medications, or various conditions such as pregnancy, liver cirrhosis, and hypothyroidism, these levels are usually well below the prolactin serum levels caused by prolactinomas [2].

The majority (> 90%) of prolactinomas are microadenomas (< 10 mm) and tend not to enlarge; only 10% of prolactinomas are macroadenomas or giant adenomas. In cases where observation alone is not an option due to clinical symptoms, medical treatment with dopamine agonists (cabergoline or bromocriptine) is effective in reducing tumor size and restoring gonadal function and fertility in most patients. Surgical tumor resection of a prolactinoma is a first-line treatment for emergencies such as pituitary apoplexy with acute visual loss. As a second-line treatment, surgical resection is indicated when medical therapy has failed as defined by tumor growth, medication side effects, or failure to control elevated serum prolactin levels. Resection of prolactinoma leads to significantly improved postoperative quality of life (QOL), whereas in the first 3–6 months after surgery QOL, and especially sinonasal symptoms transiently worsen [3, 4]. Side effects of a dopamine agonist therapy include CNS problems such as fatigue and mood changes, gastrointestinal symptoms such as nausea and constipation, and symptoms caused by vasodilation, such as nasal congestion and headache. Although the majority of patients experience side effects, these effects tend to resolve and only a small proportion of patients ultimately have to discontinue treatment due to side effects [5, 6]. More serious risks of dopamine agonists include valvular heart disease, psychosis, and impulse control disorder, but more research is needed to better understand the relationship of these complications to medical treatment [7]. Furthermore, CSF rhinorrhea can occur as a complication of medical therapy for prolactinoma in cases where large tumors have led to the destruction of the skull base and a shrinking tumor under dopamine agonists opens skull base defects with subsequent CSF leakage.

As the results of minimally invasive transsphenoidal surgery have improved, resulting in remission rates of 77–91% with low morbidity [8], the role of surgery is currently being debated and may be defined less restrictively in the future [6, 9]. In particular, the need for lifelong medical treatment and potential adverse effects may make early surgical intervention a preferable option in more cases. Radiotherapy is generally reserved for complex cases that cannot be controlled by medical therapy or surgery.

The present work aims to analyze a surgical series and to discuss the role of surgical resection versus medical therapy in the treatment of prolactinomas.

## Patients and methods

Institutional review board approval was obtained for this study. Informed consent was not required due to the retrospective and anonymous nature of this study. We analyzed a database of all patients who underwent prolactinoma surgery in our department between 2003 and 2021. Preoperative and postoperative magnetic resonance images with and without contrast were assessed. Tumor size was measured before and after surgical resection. Cavernous sinus invasion was graded according to Knosp [10]. In addition, computed tomography images were evaluated for any bony destruction by large tumors. Standard preoperative endocrinologic evaluation included serum levels of prolactin, ACTH, thyroid-stimulating hormone (TSH), free thyroxine (FT4) (thyroid function), luteinizing hormone (LH), follicle-stimulating hormone (FSH), testosterone, cortisol, growth hormone (GH), and insulin-like growth factor (IGF1). In selected cases, dynamic endocrine testing was performed to test the ability of the pituitary gland to respond to various stimuli.

The diagnosis of prolactinoma was based on the presence of a pituitary adenoma on MRI and serum prolactin levels > 150 ng/mL. Endocrine remission was defined as a postoperative serum prolactin level < 15 ng/mL.

Collected patient factors included demographics, preoperative symptoms, and prior prolactinoma treatment. Tumor factors such as Knosp grade, tumor size, and skull base destruction were evaluated. Operative technique, extent of resection, and surgical outcome and any intra- or postoperative complications were assessed. Data were collected in Excel and analyzed retrospectively. Frequencies, means, etc. were calculated using Excel.

### Surgical technique

Either a transnasal transsphenoidal approach or a transcranial approach was used for surgical resection. In the majority of cases, an endoscopic endonasal transsphenoidal approach was used. After routine preparation with perioperative antibiotics and a stress dose of hydrocortisone, the sphenoid ostia were identified on both sides and opened with rongeurs. Great care was taken not to damage the nasal mucosa, as excessive use of the bipolar, for example, can lead to postoperative complications that can significantly reduce the quality of life (QOL). The endoscope, with 0° optics, was inserted through the left nostril and fixed with a rigid arm. The anterior wall of the sella was dissected free of mucosa and opened with a high-speed drill and rongeurs. The adenoma was resected with curettes after the opening of the sella endosteum. After adenoma resection, the resulting defect was covered with hemostatic agents and fibrin glue. In cases of CSF leakage, we used fat and fascia lata for coverage, and in very rare cases of giant adenoma, a nasoseptal flap was used to close the defect. In one patient, the endoscopic transphenoidal technique was not feasible due to lateral extension of the tumor; this case was resected via a standard frontolateral approach from the right side.

## Results

### Patient characteristics

Thirty patients with prolactinoma who underwent a total of 32 procedures were identified. Prolactinomas represent 4% of all 750 pituitary adenomas which underwent surgical resection in 800 procedures at our department during the time period 2003–2021. The mean age of patients harboring prolactinomas was 37.2 ± 15.5 years (range 16–76) and 17 (56.7%) were females and 13 (43.3%) were males. The mean age of the women in our sample is 32.4 ± 11.7 (range 16–58), and the mean age of the men is 43.5 ± 17.8 (range 19–76). Table 1 provides an overview of the baseline characteristics.

**Table 1:** Baseline characteristics

	*n* (%)
Sex	
Male	13 (43.3%)
Female	17 (56.7%)
Mean age in years (±SD)	37.2 ± 15.5
Max tumor diameter	
< 1 cm	17 (56.7%)
> 1 cm	9 (30%)
> 4 cm	4 (13.3%)
Invasion of cavernous sinus	
Yes	3 (10%)
No	27 (90%)
Visual symptoms	
Yes	7 (23.3%)
No	23 (76.7%)
Reason for operation	
Apoplexy	5 (16.7%)
Adverse effects of dopamine agonists	11 (36.7%)
Insufficient response to dopamine agonists	10 (33.3%)
Progressive neurological deficit	2 (6.7%)
Patients choice	2 (6.7%)

### Preoperative diagnostics

All 30 patients received preoperative magnetic resonance imaging of the pituitary gland, and tumor size and invasion were measured. Of the 30 operated tumors, 56.7% (*n* = 17) were microadenomas, 30% (*n* = 9) were macroadenomas (≥ 10 mm), and 13.3% (*n* = 4) were giant adenomas (≥ 40 mm). In three cases, the adenoma invaded the cavernous sinus (Knosp Grade 4, 2, and 2). The chiasm was compressed in four cases. The adenoma extended into the sphenoid sinus in two cases. Despite medical therapy with dopamine agonists, 10 patients had preoperative persisting elevated prolactin levels.

### Indication for surgery

Twenty-one patients (70%) had failed medical therapy, the main reasons being intolerable side effects in 11 cases (52.4%) and insufficient response in 10 cases (47.6%). Nine patients (30%) received no medical treatment prior to surgery, of which 5 (55.6%) were operated due to pituitary apoplexy, two (22.2%) for acute visual deterioration without pituitary apoplexy and two (22.2%) patients refused medical treatment and opted for surgery as first-line treatment.

Seven patients (23.3%) had a preoperative visual impairment (four visual acuity and visual field deficits, three diplopia due to cavernous sinus invasion).

### Surgical results

**Fig. 1:**
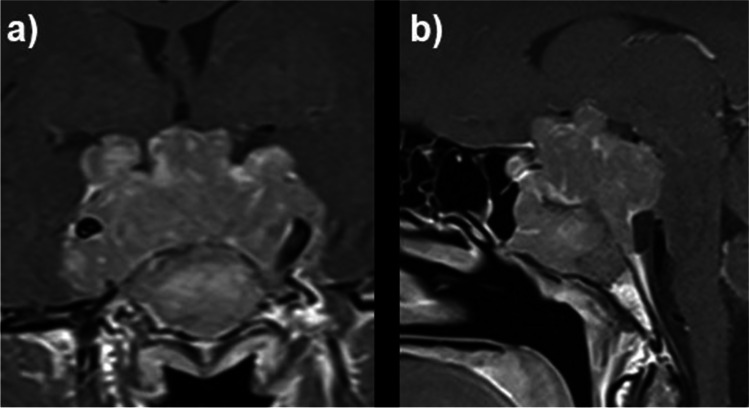
Coronal (**a**) and sagittal (**b**) MRI with contrast of a patient with a giant prolactinoma compressing the chiasm and invading the cavernous sinus on both sides, completely encasing the ICA (Knosp grade 4)

Of the 32 surgical procedures, 30 were endoscopic transsphenoidal resections (93.8%) and two were transcranial resections (6.3%) in the same patient. In this case, the endoscopic transsphenoidal technique was not feasible due to the lateral extension of the giant tumor (Fig. 1). Gross total resection (GTR) was achieved in 21 (75%) cases, and residual tumor was found in 7 (25%) cases in which postoperative imaging was available. All patients with residual tumors after surgery had an invasive growth of the prolactinoma into the cavernous sinus. An intraoperative CSF leak was reported in two cases, postoperative CSF leaks did not occur. In the case of the two transcranial surgeries with subsequent transsphenoidal tumor debulking, proton irradiation was used postoperatively, followed by dopamine agonist treatment. Mean follow-up was 34.9 ± 60.3 months (range 0–246 months). Endocrine remission was defined as a morning fasting basal PRL level of 22.3 < ng/mL and measured at the last available follow-up. Postoperative Prolactin levels were missing in three patients. The overall endocrinological remission rate after surgery was 63.3% (19 cases). A transient postoperative diabetes insipidus occurred in four patients, in two a diabetes insipidus persisted. No temporary or permanent SIADH occurred. Table 2 provides an overview of the surgical results.

**Table 2:** Surgical outcome

	*n* (%)
Extent of resection*	
GTR	21 (75%)
Residual tumor	7 (25%)
CSF leak	
Intraoperative	2 (6.7%)
Postoperative	0
Diabetes insipidus	
Transient	4 (13.3%)
Permanent	2 (6.7%)
Endocrinological remission	19 (63.3%)

*In two patients, postoperative imaging was not available

Seven patients had visual disturbances preoperatively; postoperatively, there was an objective improvement or recovery in all cases. The patient, who underwent two transcranial surgeries developed a new N. III paresis after the first surgery.

## Discussion

### Sample characteristics/preoperative diagnostics/indication for surgery

The patient characteristics of our present series are in line with other published series, with a predominance of female patients and a relatively young mean age. Primeau et al. reported a male/female ratio of 18/45 with a mean age of 31 ± 14 years [11]. Kreutzer et al. reported a male/female ratio of 79/133 with a mean age of 32 years [12] and Hofstetter et al. a male/female ratio of 13/22 with a mean age of 36.3 years [13]. Several studies have reported sex differences in prolactinoma. Due to the nature of the symptoms, men tend to be diagnosed later than women, resulting in larger tumors at presentation, which is most likely the reason why surgical series include a higher proportion of men than medical series, and why tumor characteristics and outcomes tend to be worse in male patients [14, 15].

Our series included 30% macroadenomas and 13.3% giant adenomas; this disproportional representation of tumors over 10 mm in size is also reported by other authors with rates from 47–65% of prolactinomas being larger than 10 mm in surgical series [11–13, 16].

The indication for surgery was the failure of medical therapy in 70% (*n* = 21) of cases in our series. Of those, 52.4% had failed medical therapy because of side effects, and 47.6% because of inadequate response. Of patients, 23.3% (*n* = 7) underwent surgery because of acute neurological deficits. The reasons for surgery are also similar to other published series. Primeau et al. reported drug intolerance as a reason for surgical treatment in 21% of cases, an inadequate response in 41% (*n* = 26) and acute complications of medical treatment in 16% (*n* = 10) of cases [11]. In the series of Hofstetter et al., 80% of operated patients had previously attempted medical therapy [13].

Similar to our experience, other authors reported a subgroup of patients who chose surgery as a first-line treatment: 22.2% in our series, 22% (*n* = 14) in the analysis by Primeau et al. [11], and 10.9% (*n* = 23) in the paper authored by Kreutzer et al., who also showed an increase of this group over time [12].

Due to the relatively large tumor size in our sample, the cavernous sinus was invaded in three patients and the chiasm was compressed in four patients.

### Surgical results

In line with other modern centers, we use the endonasal endoscopic transsphenoidal technique whenever possible. The overall remission rate of 63.3% is comparable to other series. Kreutzer et al. reported a remission rate of 42.7% [12], Hofstetter et al. had a rate of 70.6% in their sample [13], and Primeau et al. showed a long-term remission rate of 30% [11].

Remission rates vary widely depending on the size, invasiveness, and morphology of the adenoma; they also tend to slightly decrease during longer follow-up. In their comprehensive meta-analysis, Wright et al. reported remission rates based on tumor volume, with pooled remission rates of 76.4% for microadenomas and 47.1% for macroadenomas [17]. Numerous authors have reported that complication rates and outcomes for surgical and medical therapy are largely dependent on tumor size, with infiltrative growing tumors representing the most challenging cases [16].

The rate of gross total resection is 75% and similar to the 74.3% reported by Hofstetter et al. [18]. Surgeons have reported adverse effects of dopamine agonists on tumor resectability due to dopamine-agonist-induced tumor fibrosis. Menucci et al. reported that fibrosis is found in up to 77% of adenomas treated with dopamine agonists and may adversely affect the outcome of a surgical resection [19]. In contrast, Wright et al. reported on positive effects of a medical therapy before surgery, such as a higher postoperative remission rate [17]. Further research is needed to determine how to influence the positive and negative effects of medical therapy and the role of duration of therapy.

Several studies have identified the extent of resection (EOR) as a major predictor of postoperative hormonal remission. Thus, factors that prevent gross total resection (e.g., cavernous sinus infiltration, large tumor size with extrasellar involvement, etc.) are therefore significantly associated with lower remission rates. Other factors (not related to surgery) that significantly influence remission rates are preoperative hormone levels and preoperative medical treatment [20].

Surgical outcome is mainly determined by tumor size, invasiveness, and morphology. This has been shown in a subgroup analysis with remission rates over 80% for microprolactinomas [12].

Emergency situations and adverse effects of medical treatment may further complicate resections [19].

In our analysis, we describe a highly selected sample with only 4% of all surgically treated adenoma cases with a disproportionately high proportion of larger, invasive tumors and emergency cases. Nevertheless, the results are promising and even complicated cases can be managed by combining medical, surgical, and radiotherapy.

### Should surgery be used as a first-line treatment

Until now, surgical resection of prolactinomas has only been indicated as a first-line treatment for patients who develop progressive neurological symptoms (most commonly visual problems). Occasionally, patients refuse medical treatment and opt for surgery as a first-line treatment, as well. However, the vast majority of patients with prolactinomas are initially treated pharmacologically [21]. This is reflected by the fact that only 4% of our adenoma operations are for prolactinomas, although prolactinomas accounts for 50% of all diagnosed pituitary adenomas.

Therefore, the sample selected for surgery consists mainly of complicated cases with tumors larger than 10 mm (43.3% in our series), emergencies (23.3% in our series), and cases that failed medical treatment (70% in our series), which are all aspects that increase the risk of complications and worsen the outcome.

Studies that have analyzed the surgical outcome of microprolactinomas show better results (compared to macroprolactinomas, e.g., heterogeneous groups) with long-term remission rates of 70–100% [16, 22]. The complication rates of prolactinoma surgery range from 0 to 5% [8, 12, 23]. Another important factor to consider is the evolution of both microscopic and endoscopic techniques in recent years. New sophisticated instruments, neuronavigation, and high-resolution cameras have led to an overall improvement in outcomes. Although randomized controlled trials comparing endoscopic and microscopic technique do not show a significant advantage of either technique, several authors have reported advantages of the endoscopic approach due to better visualization especially in tumors with parasellar extension, leading to higher gross tumor removal and lower morbidity. Regardless of the technique, surgeon experience seems to be a more relevant factor [24, 25].

Based on the good results, the question of whether the indication for surgery for prolactinomas should be reevaluated and whether minimally invasive transsphenoidal surgery should be offered as a first-line treatment option (especially for microprolactinomas) has been increasingly discussed by physicians involved in the treatment of prolactinomas in recent years [6, 7, 9, 17, 22, 26, 27].

An important aspect fueling this discussion is that patients with prolactinomas often require lifelong medical treatment, as recurrences have been shown to occur in 54% once medical therapy is discontinued [18]. While there is uncertainty regarding the long-term effects of medical treatment, side effects are reported in 15–42% of patients and primary or secondary resistance is common, with surgery required as a second-line treatment in 14–38% of cases [5, 28].

A comparative study including 70 patients with prolactinomas by Park et al. showed better outcomes in terms of dopamine agonist-free remission for the group that was treated surgically [26]. However, to determine the optimal treatment for each individual patient, prospective trials are needed to clarify whether surgical treatment of prolactinoma as first-line therapy or after a short course of medical therapy is a justified option with respect to treatment side effects or treatment-related morbidity.

### Limitations

The small sample size as well as the relatively short/respectively varying follow-up time in combination with the retrospective nature of the study reduce the generalizability of our data.

## Conclusion

Our analysis describes a highly selected sample with a disproportionate number of larger, invasive tumors and emergency cases. Nevertheless, the results are satisfactory and comparable with other published surgically treated series. The consistently good results of transphenoidal surgery, especially for microprolactinomas, have led to a greater acceptance of surgery in the treatment of prolactinomas in recent years. The timing of surgery in each individual case must be determined by a multidisciplinary team to ensure the best possible outcome.

## Data Availability

All of the material is owned by the authors and no permissions are required.
